# Sympathetic nerves control bacterial clearance

**DOI:** 10.1038/s41598-020-72008-4

**Published:** 2020-09-14

**Authors:** Yugeesh R. Lankadeva, Clive N. May, Michael J. McKinley, Melanie R. Neeland, Shuai Ma, Dianna M. Hocking, Roy Robins-Browne, Sammy Bedoui, David G. S. Farmer, Simon R. Bailey, Davide Martelli, Robin M. McAllen

**Affiliations:** 1 Florey Institute of Neuroscience and Mental Health, University of Melbourne, 30 Royal Parade, Parkville, VIC 3052 Australia; 2 Murdoch Children’s Research Institute, Parkville, VIC Australia; 3 Department of Microbiology and Immunology, University of Melbourne At the Peter Doherty Institute for Infection and Immunity, Melbourne, VIC Australia; 4 Faculty of Veterinary Science, University of Melbourne, Parkville, VIC Australia; 5 Department of Biomedical and NeuroMotor Sciences, University of Bologna, Bologna, Italy

**Keywords:** Neuroimmunology, Peripheral nervous system, Medical research

## Abstract

A neural reflex mediated by the splanchnic sympathetic nerves regulates systemic inflammation in negative feedback fashion, but its consequences for host responses to live infection are unknown. To test this, conscious instrumented sheep were infected intravenously with live *E. coli* bacteria and followed for 48 h. A month previously, animals had undergone either bilateral splanchnic nerve section or a sham operation. As established for rodents, sheep with cut splanchnic nerves mounted a stronger systemic inflammatory response: higher blood levels of tumor necrosis factor alpha and interleukin-6 but lower levels of the anti-inflammatory cytokine interleukin-10, compared with sham-operated animals. Sequential blood cultures revealed that most sham-operated sheep maintained high circulating levels of live *E. coli* throughout the 48-h study period, while all sheep without splanchnic nerves rapidly cleared their bacteraemia and recovered clinically. The sympathetic inflammatory reflex evidently has a profound influence on the clearance of systemic bacterial infection.

## Introduction

The innate immune system plays a major role in the body’s defense against acute infections^1^. Initially, this involves recognition of pathogen-associated molecular patterns that trigger acute inflammatory responses^2^. There then follows a sequence of molecular and cellular events that work towards eliminating the invading pathogen and returning the body to a normal healthy state^2^.

There is strong evidence that the central nervous system actively modulates inflammatory processes^3^. It does this by activating at least two anti-inflammatory effector mechanisms: the hypothalamic-pituitary axis and autonomic neural pathways^3^. Systemic inflammation is most commonly evoked experimentally by intravenous administration of lipopolysaccharide to cause an acute, sterile endotoxaemia. In such a model in rats, the inhibitory influence of autonomic neural pathways is revealed by loss of function when the splanchnic sympathetic nerves are cut: the circulating levels of pro-inflammatory cytokines, including tumor necrosis factor alpha (TNF-α), interleukin (IL)-6 and interferon gamma (IFN-γ), are strongly enhanced, while those of the anti-inflammatory cytokine IL-10 are decreased^4,5^. These changes occur in the absence of any difference in circulating glucocorticoid levels^5^. This coordinated anti-inflammatory action is activated endogenously as a reflex response to the inflammatory challenge itself, and is mediated by the splanchnic nerves^4–7^. In rats, the anti-inflammatory action lasts for at least 6 hours^4^. What has not yet been established, however, is how the anti-inflammatory action of this reflex affects the body’s ability to combat a bacterial infection. To this end we compared the effect of prior splanchnic nerve section (or a sham operation) on the disease course of a well characterized large animal model of septicaemia: the conscious sheep infused with a bolus of live *Escherichia coli* (*E. coli*) bacteria.

## Results

### *E. coli* bacteraemia

**Figure 1:**
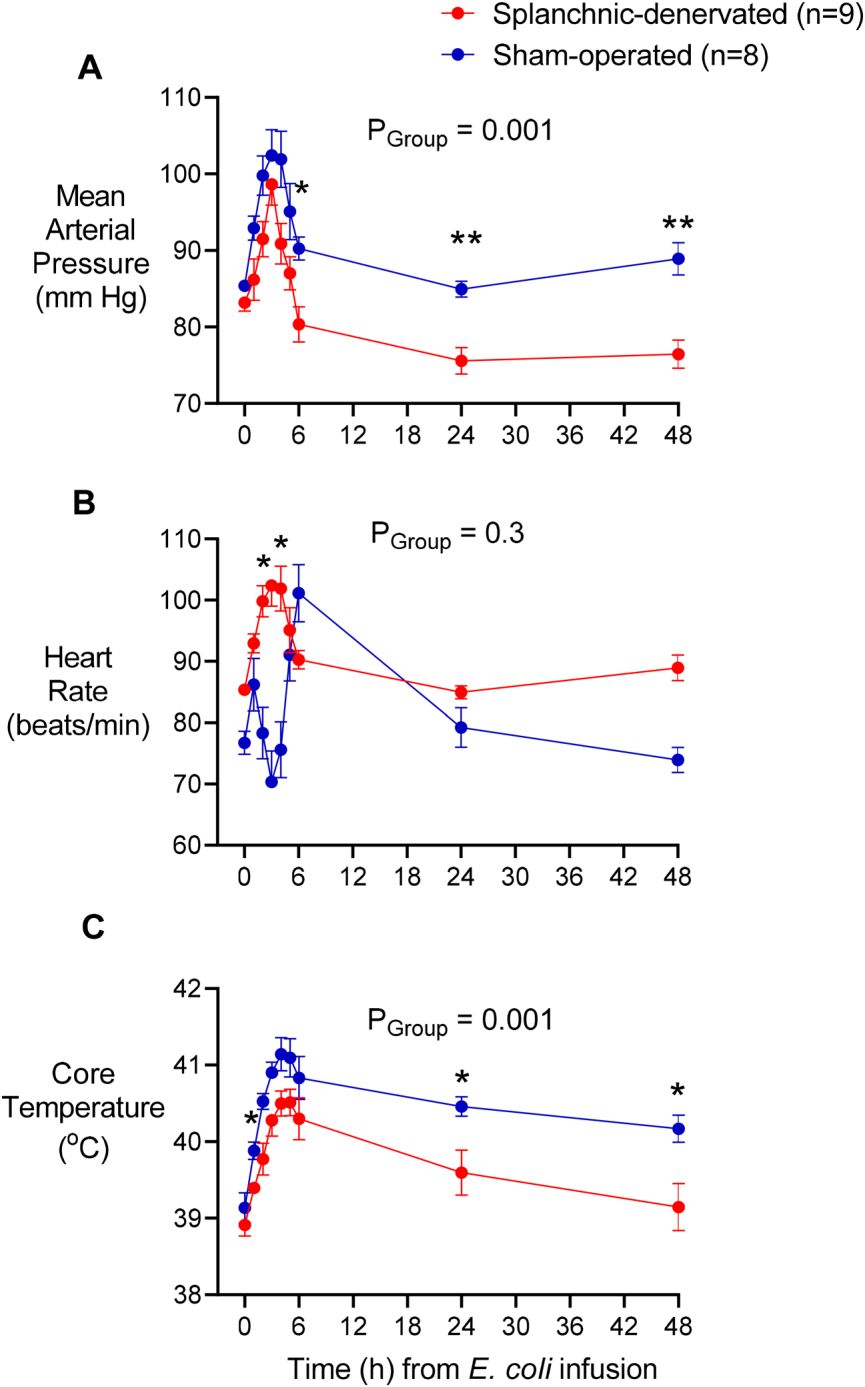
Systemic hemodynamics in response to *Escherichia coli* in conscious sheep following bilateral splanchnic denervation or sham surgery. Mean arterial pressure (**A**), heart rate (**B**) and core temperature (**C**) after infusion of live *E. coli* at time 0. Data are mean ± sem. Time 0 is the mean of the 12th hour of the baseline period, and times 1.5–48 are means of 30-min periods. *P* values represent experimental group effects (Splanchnic or Sham denervation) from a two-way repeated measures analysis of variance from 0 to 48 h after inoculation with *E. coli*. *P < 0.05, **P < 0.01 and ***P < 0.001 indicates significant differences between Splanchnic denervated animals and sham-operated sheep using a Bonferroni post hoc comparison.

As shown previously^8^, infusion of live *E. coli* into conscious sheep caused an initial increase in mean arterial pressure that peaked around 3 h, followed by a slow decrease over the following 24 h (Fig. 1A). The mean arterial pressure in splanchnic-denervated animals tracked ~ 5–10 mmHg lower than in intact animals (Fig. 1A), as would be expected after the removal of vasomotor tone from a major vascular bed. Heart rate increased in both groups (though after an initial bradycardia in sham-operated animals; Fig. 1b). Arterial blood lactate doubled in both groups, though it peaked earlier in the splanchnic-denervated animals before declining (Table 1). All sheep developed a fever, but this was less, and shorter-lasting in the splanchnic-denervated animals (Fig. 1C). As expected from denervation of the adrenal medulla, plasma catecholamine levels, especially adrenaline, remained low after splanchnic nerve sections (Table 1). No animal in either group died before completion of the experiment.

**Table 1:** Arterial blood gases, blood lactate and plasma catecholamines in response to bolus infusion of live *Escherichia coli* in conscious sheep following bilateral splanchnic denervation or sham surgery.

	0 h	1.5 h	3 h	6 h	24 h	48 h
**Arterial PO**_**2**_** (mmHg)**						
Splanchnic denervation (n = 9)	99 ± 2	94 ± 3	96 ± 4	98 ± 7	99 ± 3	101 ± 3
Sham-operated (n = 8)	101 ± 3	94 ± 2	92 ± 2	91 ± 4	95 ± 3	98 ± 2
**Arterial PCO**_**2**_** (mmHg)**						
Splanchnic denervation (n = 9)	33 ± 1	32 ± 2	31 ± 1	31 ± 1	30 ± 1	33 ± 1
Sham-operated (n = 8)	33 ± 1	31 ± 2	31 ± 2	29 ± 2	31 ± 2	33 ± 1
**Arterial Blood Lactate (mmol/L)**						
Splanchnic denervation (n = 9)	0.36 ± 0.03	0.68 ± 0.23	1.32 ± 0.65	1.03 ± 0.14	0.44 ± 0.07	0.41 ± 0.05
Sham (n = 8)	0.42 ± 0.05	0.70 ± 0.09	0.69 ± 0.08	1.16 ± 0.21	0.64 ± 0.11	0.55 ± 0.10
**Plasma Noradrenaline (ng/mL)**						
Splanchnic denervation (n = 6)	0.14 ± 0.03	0.22 ± 0.09		0.07 ± 0.02	0.21 ± 0.07	0.16 ± 0.05
Sham (n = 6)	0.75 ± 0.17*	0.62 ± 0.17		0.57 ± 0.12*	0.76 ± 0.15*	0.86 ± 0.21
**Plasma Adrenaline (ng/mL)**						
Splanchnic denervation (n = 6)	0.03 ± 0.01	0.06 ± 0.01		0.08 ± 0.02	0.06 ± 0.01	0.07 ± 0.02
Sham (n = 6)	0.24 ± 0.04 **	0.20 ± 0.03*		0.21 ± 0.03*	0.26 ± 0.05*	0.25 ± 0.03***

Values are between-animal mean ± sem.*P < 0.05 **P < 0.01 and **P < 0.001 indicates between-group significant differences between sheep following bilateral splanchnic denervation (n = 9) compared with sham surgery (n = 8) at 0, 1.5, 3, 6, 24 and 48 h after infusion of a bolus of *E. coli* using a Sidak’s multiple comparisons test.

### Plasma cytokines

**Figure 2:**
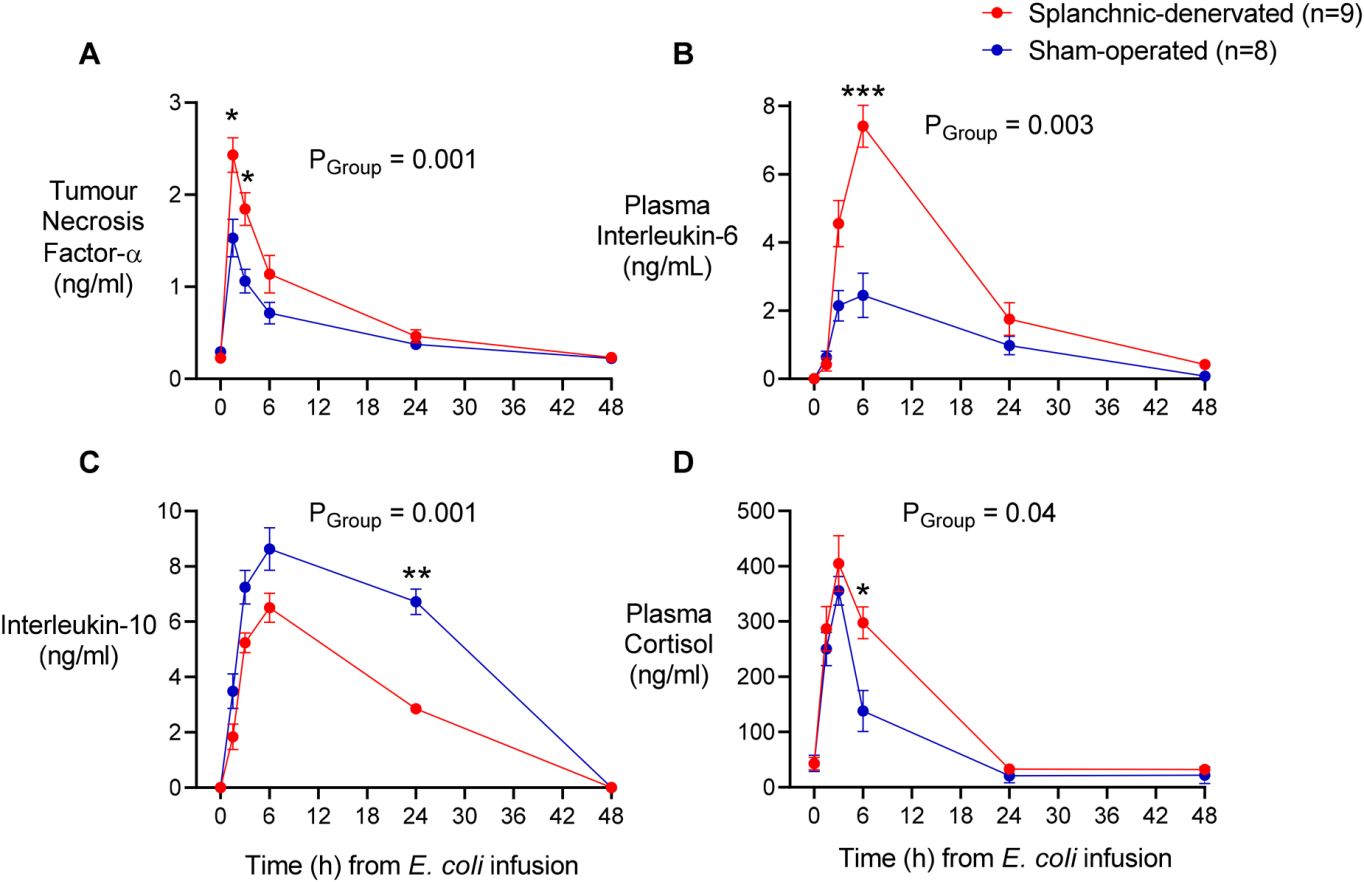
Plasma inflammatory and anti-inflammatory cytokine and cortisol responses to *Escherichia coli* in conscious sheep following bilateral splanchnic denervation or sham surgery. Tumor necrosis factor α (**A**), interleukin-6 (**B**), interleukin-10 (**C**) and cortisol (**D**) after infusion of live *E. coli* at time 0. Data are mean ± sem. Time 0 is the mean of the 12th hour of the baseline period, and times 1.5–48 are means of 30-min periods. *P* values represent experimental group effects (Splanchnic or Sham denervation) from a two-way repeated measures analysis of variance from 0 to 48 h after inoculation with *E. coli*. *P < 0.05, **P < 0.01 and ***P < 0.001 indicates significant differences between Splanchnic denervated animals and sham-operated sheep using a Bonferroni post hoc comparison.

As in rodents challenged with LPS derived from *E. coli*^4,5^, the response of sheep to live *E. coli* was characterised by a large early rise in plasma TNF-α, followed by a slower, more prolonged rise in plasma IL-6. Levels of the anti-inflammatory cytokine IL-10 followed a similar time course (Fig. 2): all returned to normal by 48 h (Fig. 2). As also established for rodents, splanchnic denervation enhanced plasma levels of TNF-α and IL-6, while reducing plasma IL-10: evidence that a coordinated anti-inflammatory reflex response had been disabled^5,9^.

### Bacterial clearance

**Figure 3:**
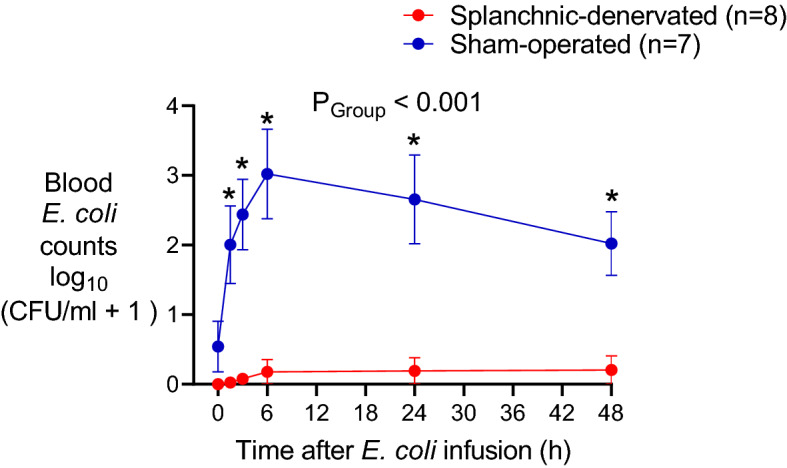
Blood bacterial counts in response to an infusion of *Escherichia coli* in conscious sheep following bilateral splanchnic denervation or sham surgery. Gram-negative colony forming units found in arterial blood after infusion of live *E. coli* at time 0. Data are mean ± sem. Time 0 is the mean of the 12th hour of the baseline period, and times 1.5–48 are means of 30-min periods. *P* values represent experimental group effects (Splanchnic or Sham denervation) from a two-way repeated measures analysis of variance from 0 to 48 h after inoculation with *E. coli*. *P < 0.05, **P < 0.01 and ***P < 0.001 indicates significant differences between Splanchnic denervated animals and sham-operated sheep using a Bonferroni post hoc comparison.

Remarkably, splanchnic denervation had a profound influence on bacterial clearance (Fig. 3). In sham-operated animals, blood *E. coli* counts rose rapidly after infusion; peaking after 6–24 h (Fig. 3). Blood levels > 10,000 colony forming units (CFU)/ml were measured in 5 of 8 sham-operated animals. In contrast, all 7 splanchnic-denervated animals showed no, or minimal (only one sheep < 50 CFU/ml), blood *E. coli* counts from 1.5 h onwards (Fig. 3). This difference could not be explained by plasma cortisol levels, which were actually greater in the splanchnic-denervated animals (Fig. 2).

### Circulating leukocytes

**Figure 4:**
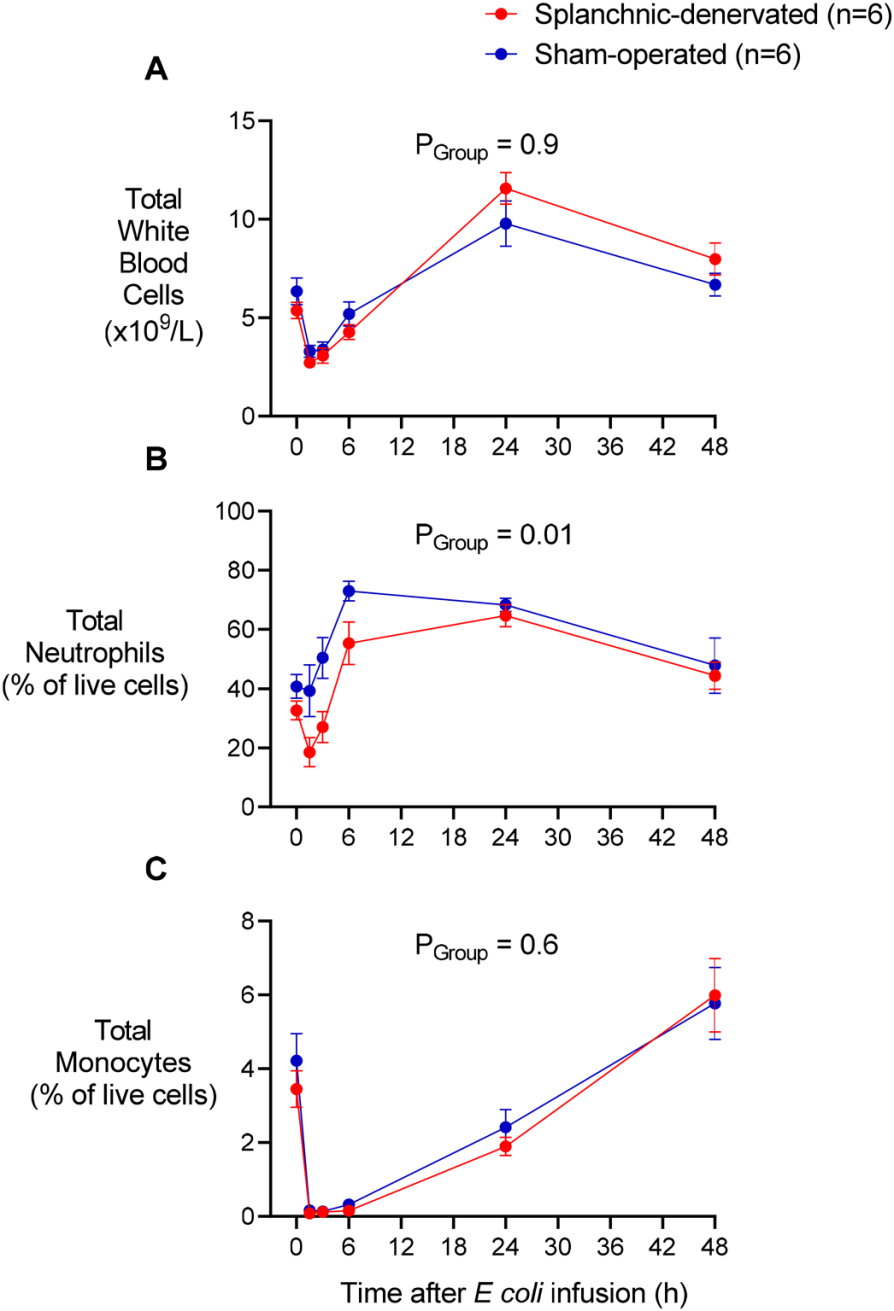
Total white blood cells and leukocyte subsets in response to *Escherichia coli* in conscious sheep following bilateral splanchnic denervation or sham surgery. Total white blood cells (**A**), total neutrophils (**B**) and total monocytes (**C**) after infusion of live *E. coli* at time 0. Data are mean ± sem. Time 0 is the mean of the 12th hour of the baseline period, and times 1.5–48 are means of 30-min periods. *P* values represent experimental group effects (Splanchnic or Sham denervation) from a two-way repeated measures analysis of variance from 0 to 48 h after inoculation with *E. coli*. *P < 0.05, **P < 0.01 and ***P < 0.001 indicates significant differences between Splanchnic denervated animals and sham-operated sheep using a Bonferroni post hoc comparison.

Circulating leukocytes in the two animal groups behaved similarly, except for a pronounced reduction in neutrophils of splanchnic-denervated sheep over the first 3 h (Fig. 4B). Thereafter, neutrophil levels were raised in both sets of animals. Total white cells in both groups showed an acute fall followed by a delayed rise (Fig. 4A). In both groups also, monocytes disappeared from the blood by 1.5 h; then recovered gradually over the next 24-48 h (Fig. 4C), mirroring what is seen in experimental endotoxaemia in humans^10^.

## Discussion

Here we report that reflex neural influences, mediated by the splanchnic sympathetic nerves, have a profound effect on the resolution of sublethal *E. coli* bacteraemia. Animals with cut splanchnic nerves mounted a stronger acute inflammatory response than animals with intact nerves, characterised by higher circulating levels of the pro-inflammatory cytokines, TNF-α and IL- 6, and lower levels of the anti-inflammatory cytokine IL-10. This coordinated pro-inflammatory shift matched that seen in endotoxaemic rats after acute splanchnic nerve section^4,9^, indicating a basic biological response that transcends species. The greater pro-inflammatory response in splanchnic-denervated animals most likely contributed to their enhanced ability to clear bacteraemia. In the present study, plasma cortisol levels were not lower in the splanchnic-denervated animals (in fact they were higher), so this factor could not account for their stronger inflammatory response to a systemic *E. coli* infection. A direct neural anti-inflammatory action is thus implicated, and the present findings show its importance in the context of an acute systemic infection.

Despite the removal within minutes of bacteria from the blood of the splanchnic-denervated animals, their enhanced inflammatory cytokine profile outlasted the presumed stimulus by about a day (Fig. 2). We may therefore expect the consequences of this shift for innate immune function to last for at least that long. Interestingly, the greater inflammatory cytokine response in sheep with cut splanchnic nerves did not result in a higher fever. In fact, fever was lower and briefer. This shows that the fever in these animals was not driven primarily by circulating TNF-α or IL-6. It seems likely that the persistent bacteraemia in sham-operated animals was a major factor driving their enhanced, prolonged fever, but unmeasured cytokines could have contributed. In this context, we may note the finding in rats that lipopolysaccharide-stimulated levels of the major pyrogenic cytokine, IL-1ß^11^ were actually reduced in animals with cut splanchnic nerves^4^, (against the pattern for proinflammatory cytokines).

Flow cytometry revealed one important difference between animals with and without splanchnic nerves. In the first 3 h after infection, there was a pronounced fall in circulating neutrophils (reduced percentage of a reduced white cell count) that was deeper in splanchnic-denervated sheep. That fall likely reflects removal of activated neutrophils from the circulating pool as a result of adhesion to endothelial cells^12^. The stronger inflammatory cytokine response of splanchnic-denervated animals evidently enhanced this process. Additionally, the lack of circulating adrenaline in these animals might have contributed to the difference, by removing beta receptor-mediated inhibition of neutrophils^13^. Thereafter, circulating neutrophil levels rose as expected in a bacterial infection, and did so with a similar trajectory in the two sets of animals. Monocytes also disappeared rapidly from the circulating pool to peripheral tissues, although their loss and subsequent replenishment were not different between splanchnic-denervated and sham-operated sheep.

The lower blood pressure of splanchnic-denervated animals in the face of vasodilator cytokines was expected, given that a major vascular bed (the splanchnic) had been disconnected from its neural vasomotor drive, preventing compensatory vasoconstriction. The hypotension seems unlikely to be a cause of improved bacterial clearance. The differences in heart rate between the two animal groups were not expected. The early bradycardia seen in the sham-operated sheep was almost certainly a vagal baroreflex response to the early peak in blood pressure. Yet this was absent from the denervated sheep. It seems most likely that some factor(s) (e.g. their stronger inflammatory cytokine profile) suppressed their cardiac vagal tone and vagal baroreflex. This then allowed the increased sympathetic drive to the heart (which we have measured directly in this ovine model^8^) to dominate. Such suppression has been reported to occur in humans injected with low doses of lipopolysaccharide^14^, although not in rats^15^.

We previously demonstrated in rats that the splanchnic nerve-mediated anti-inflammatory action is distributed across the abdominal organs innervated by that sympathetic nerve, including the spleen, intestines, stomach, pancreas, liver and adrenal medulla^16^. While the splanchnic nerves drive the release of catecholamines from the adrenal medulla^17^, the reflex survives adrenalectomy, indicating that direct release of neurotransmitters from sympathetic nerve terminals suppresses inflammation^16^. We infer that removal of the braking influence of the splanchnic nerves enhances the capacity to sequester and kill circulating bacteria by phagocytic and cytotoxic cells distributed broadly in the abdominal viscera. In addition, we infer that the stronger circulating pro-inflammatory cytokine profile of denervated animals would have enhanced phagocytosis by cells distant from nerves, such as neutrophils^12^. Their greater early sequestration from the circulating pool likely reflects this.

### Strengths and limitations

In the present study, the disappearance of live bacteria from the circulation in splanchnic-denervated sheep was extremely rapid (within 1.5 h). It should be borne in mind that these animals had all experienced a month without functional splanchnic nerves, and consequently a lack of circulating adrenaline, which might have affected their inflammatory responsiveness. This seems unlikely to be the main explanation, however, because the same pattern of enhanced TNF-α and IL-6 with attenuated IL-10 responses to inflammatory stimulation was seen in rats after acute splanchnic nerve section^5,9^.

Caution is needed before extrapolating from these findings. The present study used a single bolus infusion of live bacteria, rather than a sustained infection leading to sepsis with end organ damage^18–20^; the optimal balance between pro- and anti-inflammatory host responses may be different between those conditions, and that difference may be time-dependent. Also, this study only investigated systemic infection with one Gram–negative bacterial species, albeit one that is the commonest cause of Gram-negative sepsis in humans; different rules may govern how nerves interact with immune responses to other infectious agents such as viruses, fungi or Gram-positive bacteria^21^, or when the inflammation is localised rather than systemic^22^.

### Significance

We speculate that this neurally-mediated action is relevant in clinical sepsis, where an initial hyperinflammatory state is typically followed 1–3 days later by a phase of immunosuppression^23,24^. During the early hyperinflammatory phase, neural inhibition of inflammation should be helpful in reducing the damaging effects of inflammatory mediators on host tissues. But if the neural influence persists for days rather than hours, as may be expected if the infection that drives it has not been cleared, it could impede innate immune defenses against pathogens. This neural reflex may thus be a factor that contributes to the delayed phase of immunosuppression in sepsis^23,24^. Carefully timed interventions to block the reflex (e.g. giving β_2_-adrenoreceptor antagonists or local anaesthesia of the splanchnic nerves) might therefore have therapeutic benefit. It may also be worth considering prophylactic enhancement of innate immunity by reducing the neural inhibition of inflammation, for example, before surgical procedures with a high risk of infection.

## Materials and methods

### Animal preparation

All protocols were approved by the Animal Ethics Committee of the Florey Institute of Neuroscience and Mental Health under guidelines of the National Health and Medical Research Council of Australia. Merino ewes (35–45 kg bodyweight) were housed in individual metabolic cages with free access to 5 L of water and 800 g of oaten chaff daily.

Sheep underwent two preliminary aseptic surgical procedures under general anesthesia induced with 15 mg/kg sodium thiopental (JUROX, Rutherford, Australia) and, after intubation, maintained on 2.0–2.5% isoflurane (ISOFLO, Zoetis, Rhodes, Australia). The splanchnic sympathetic nerves on each side were exposed retroperitoneally through flank incisions. Animals in the two experimental groups were alternated to receive either bilateral splanchnic denervation (Splanchnic denervation, 9 sheep) or a sham surgical procedure (splanchnic nerves exposed but not injured; sham-operated, 8 sheep). The wounds were sutured in three layers, and animals allowed to recover, during which time no adverse effects were detected in either group. Three to four weeks later, a brief second surgical procedure prepared the animals for experimentation. In this, the left carotid artery was cannulated for measurement of arterial pressure and the left jugular vein was cannulated for infusion of live *E. coli* and fluids. A thermocouple was inserted into the neck alongside the left carotid artery for measurement of core temperature. Animals were given analgesia (50 mg flunixin meglumine; FLUNIXON, Norbrook, Tullamarine, Australia) and procaine penicillin (900 mg i.m. ILIUM, Troy Laboratories, Glendinning, Australia) during surgery and then 24 h and 48 h postoperatively after the first and second surgical procedures. Animals were allowed 5 days of recovery from the second surgical procedure before experimentation. A pressure transducer attached to the arterial cannula was used to record arterial pressure. Arterial pressure and core temperature (°C) were recorded on a computer using a CED MICRO 1,401 interface and Spike 2 software (Cambridge Electronic Design, Cambridge, UK). Heart rate was derived from the arterial pressure signal.

### Experimental protocol

Apart from a single Splanchnic denervation animal (the first), experiments were performed on two sheep at a time: one Splanchnic-denervated paired with one Sham-operated animal. After 12 h of baseline measurements, bacteraemia was induced in conscious sheep by an intravenous bolus infusion of live *E. coli* (2.8 × 10^9^ colony-forming units over 30 min). *E. coli* was cultured from stock derived from a human septic patient, as used in previous studies in this laboratory^18–20,25,26^. Following infusion of *E. coli*, all sheep received an infusion of isotonic sodium chloride (Baxter, Old Toongabbie, NSW, Australia; 1 mL^−1^ kg^−1^ h^−1^) as fluid supplementation. Arterial pressure and core temperature were monitored over the following 48 h. No antibiotics or catecholamines were administered. At the end of the experiments, animals were killed with an intravenous overdose of sodium pentobarbital (LETHABARB, Virbac, Wetherill Park, Australia).

### Blood samples

Arterial blood samples were collected at the end of the 12 h baseline period and then at 1.5, 3, 6, 24, and 48 h following the infusion of live *E. coli.* Measurements were made of plasma cytokines, cortisol, catecholamines, blood gases, blood bacterial cell counts, and total white blood cell counts; flow cytometry analysis was also performed. Plasma TNF-α was measured using a commercially available enzyme-linked immune-sorbent (ELISA) kit (Kingfisher Biotech Inc., MN, USA). Plasma IL-6 and IL-10 levels were assayed using in-house ELISAs, as previously described^19,20,26^. Plasma cortisol, adrenaline and noradrenaline concentrations were measured using commercially available ELISA kits (Abnova, Taipei City, Taiwan). Arterial blood gases and lactate were determined using a radiometer (ABL systems 625, Copenhagen, Denmark). Total white cell counts in blood were determined using a HEMOCUE (Radiometer Pacific P/L, Mt Waverley, Vic., Australia).

For flow cytometry, 100µL of heparinised whole blood was lysed with 1 mL of red cell lysis buffer for 10 min at room temperature. Cells were washed three times with 2 mL FACS buffer (2% heat inactivated horse serum, 2 mM EDTA in PBS) and centrifuged at 600 x*g* for 5 min at 4 °C. All samples were prepared for flow cytometry by resuspending 3 × 10^6^ cells in 25 µL FACS buffer and then adding 25µL of surface marker antibody mixes (made up at 2 ×) for 30 min on ice. Following staining, cells were washed twice with 2 mL FACS buffer and centrifuged at 600×*g* for 5 min at 4 °C and resuspended in 150 µL FACS buffer for acquisition. The antibodies used were anti–MHC class II (MHC II)–Pacific Blue (clone 49.1; locally produced), anti-CD14-A700 (clone TUK4), anti-CD172a (SIRPα) (clone DH59B), anti-CD16 FITC (clone KD1), anti-CD8 FITC (clone 38.65), anti-CD4 A647 (clone 44.38) anti-CD5R (clone 20.96), and anti-mouse IgG1 coupled to phycoerythrin. Propidium iodide was used as a viability dye. Flow cytometry was performed using a BD LSRFortessa X-20 and analysed using FlowJo software v10.6. Monocytes were defined as MHCII^int^, SIRPα^+^, CD14^+^, Neutrophils were defined as MHCII^low^ SIRPα^+^ SSC^high^, and lymphocytes as FSC^low^, SSC^low^, SIRPα^-^ cells, based on our previous work and phenotypes reported in the literature for ovine immune cells^27,28^.

Enumeration of *E. coli* in the blood was determined using a colony-forming assay^29^. Blood samples were serially diluted 1 in 10 in sterile PBS. A total volume of 1.0–1.6 ml of undiluted and diluted blood was spotted in 20 μl drops onto Luria–Bertani agar plates incubated in air for up to 48 h at 37 °C. Colonies were counted and expressed as CFU/ml.

### Statistical analysis

Data are expressed as mean ± standard error of the mean. The number of observations for each variable (n) is shown in each table or figure, respectively. Mean arterial pressure, heart rate and core temperature are presented as absolute values as 30-min averages over the 12th hour of baseline and at pre-defined time intervals after induction of bacteraemia. After a two-way repeated measures analysis of variance, post hoc comparisons were made by the Bonferroni test. Factors were experimental group and time. Analyses were performed using GraphPad PRISM (GraphPad Software, La Jolla, CA, USA). *P* values from within-subjects were conservatively adjusted using the Greenhouse–Geisser method. Two-sided *P* ≤ 0.05 was considered statistically significant.
